# Pharmacy and Travel Medicine: A Global Movement

**DOI:** 10.3390/pharmacy7020039

**Published:** 2019-04-24

**Authors:** Larry Goodyer

**Affiliations:** School of Pharmacy, De Montfort University, The Gateway, Leicester, LE1 9BH, UK; lgoodyer@dmu.ac.uk

This is the first special edition of a journal that has focused specifically on Pharmacy Practice and travel medicine. Pharmacist involvement in delivering travel health services is a relatively new phenomenon and if a call had gone out for similar publications just ten years ago, there would have been very few takers. Contained in this edition are a range of articles that examine current practice by pharmacists in delivering a travel medicine service, with some clearly describing how such services have developed. Some of these articles have been written by the committee members of the International Society of Travel Medicine (ISTM) Pharmacists Professional Group and I would encourage all pharmacists with an interest in this new discipline to join ISTM. Looking at these papers, there does seem to be a common thread in the process by which pharmacist involvement has grown so rapidly.

Undoubtably, there is a long and somewhat uncharted history whereby community-based pharmacists have offered informal advice and support to the travelling public. Many will visit a pharmacy before departure for travel-related medicines and other health products, whereby a pharmacist might be approached for advice. However, it is only recently that they have offered full services that include consultations and vaccinations. In many countries, the development has begun with changes in legislation and policy to allow pharmacists to administer influenza vaccines as part of national immunization programmes. Alongside this has been the introduction of mechanisms that allow pharmacists to supply prescription-only medicine, either by special protocols, gaining limited prescribing authority, or in some cases, full prescribing authority. It was not long after the introduction of influenza pharmacy programmes that pharmacists then began to offer other vaccination services, including those associated with travel. In some regions, community pharmacists can also offer prescription items for travel, including antimalarials and antibiotics, as prescribers themselves or under protocol. Even community pharmacy premises have undergone changes, with most now having a consultation room in which such clinical services can be delivered. It will not be long before the pharmacist becomes as much associated with the consultation area as the dispensary.

As always, with such rapid changes, there could be potential challenges and issues that may need be addressed. An important consideration is the further training of pharmacists to deliver such services. The immunization technique is not taught as part of the preregistration/undergraduate curriculum in all regions where pharmacists undertake such activities. Robust training and assessment of these skills should be undertaken post-registration and competence should be updated regularly. In addition, Travel Medicine is becoming a specialty in its own right and pharmacists should be prepared to engage with the necessary education and training required to deliver a safe and effective service.

There is a potential issue concerning pharmacists who do not give various vaccines on a regular basis in terms of maintaining their competence. Further, there is an important distinction between offering a vaccination supply and administration service and a full travel health service. The latter requires a comprehensive risk assessment of travelers and constructing a management plan that could take a considerable amount of time for those with complex itineraries and/or special needs. This implies not only a call on the pharmacist’s time from other duties, but a higher level of training and competence. It could be argued that the vast majority of the traveling public do not require such comprehensive consultations, e.g., they go on lower-risk short holidays in resorts where perhaps one or two vaccines have been recommended, and the pharmacist may not need extensive further training. However, those travelers at a greater risk should ideally be referred to a more highly trained and experienced pharmacist or another health professional. It is uncertain as to whether such referrals will take place in the community pharmacy environment.

To date, there does seem to be a good level of satisfaction amongst users of pharmacy services and perhaps a continued rise in provision will raise awareness amongst the traveling public to seek advice. Further work is needed to identify the training needs, models of delivery, and effectiveness of this new pharmacy activity.

